# Factors Associated With Uncontrolled Blood Pressure and Its Association With Cardiovascular and All-Cause Mortality Among Adults Receiving Antihypertensive Therapy: A Retrospective Cohort Study Using NHANES 2013-2018 Linked Mortality Data

**DOI:** 10.7759/cureus.117143

**Published:** 2026-09-29

**Authors:** Emmanuel Soyombo, Oluleke A Adeyanju, Christabel A Dike, Chukwuka S Umemba, Chima Iloanugo, Onyedikachi M Amagwu, Chiagozie Ezedinachi, Chiamaka N Ukpabi

**Affiliations:** 1 Internal Medicine, Sandwell and West Birmingham Hospitals NHS Trust, Birmingham, GBR; 2 General Practice, Ladoke Akintola University of Technology, Ogbomoso, NGA; 3 Internal Medicine, My Medical PC, New York, USA; 4 Family Medicine, Medical College of Wisconsin, Milwaukee, USA; 5 Internal Medicine, Maidstone and Tunbridge Wells NHS Trust, Maidstone, GBR; 6 Psychiatry and Behavioral Sciences, American University of Barbados, St. Michael, BRB; 7 Family Medicine, Western University, London, CAN; 8 Internal Medicine, University Hospital Southampton NHS Foundation Trust, Southampton, GBR

**Keywords:** antihypertensive therapy, blood pressure control, health disparities, hypertension, mortality, nhanes

## Abstract

Background: Uncontrolled blood pressure remains common among adults in the United States receiving antihypertensive therapy. However, contemporary nationally representative evidence examining associated factors and subsequent mortality within the same treated population remains limited.

Objective: To identify factors associated with uncontrolled blood pressure and examine its association with all-cause and heart disease mortality.

Methods: This retrospective cohort study used National Health and Nutrition Examination Survey (NHANES) 2013-2018 data linked to mortality through 2019. Adults receiving antihypertensive therapy with complete data were included. Uncontrolled blood pressure was defined as mean systolic pressure of at least 130 mmHg or diastolic pressure of at least 80 mmHg. Survey-weighted logistic and Cox regression were performed.

Results: The sample included 3,311 adults, representing an estimated 42,895,551 US adults after application of the NHANES examination weights. Uncontrolled blood pressure affected 56.9%. Higher odds were found among other Hispanic adults (adjusted odds ratio (AOR) 1.50, 95% confidence interval (CI), 1.08-2.07), non-Hispanic Black adults (AOR 1.63, 95% CI, 1.29-2.05), and non-Hispanic Asian adults (AOR 1.68, 95% CI, 1.15-2.45). Uncontrolled blood pressure was not significantly associated with all-cause mortality (adjusted hazard ratio (HR) 1.24, 95% CI, 0.87-1.78) or heart disease mortality (adjusted HR 0.97, 95% CI, 0.51-1.84).

Conclusion: Uncontrolled blood pressure remained common and differed across population groups. Future studies should use repeated measurements, objective treatment data, and longer follow-up.

## Introduction

Hypertension remains a leading modifiable risk factor for cardiovascular disease (CVD), stroke, chronic kidney disease, and premature mortality [1,2]. Despite advances in detection and treatment, hypertension remains common in the United States. Its age-adjusted prevalence was 32.9% in 2017-2020 [3]. Sustained blood pressure (BP) control remains a public health challenge [3]. Pharmacological BP lowering reduces the risk of major cardiovascular events and mortality [1,4]; however, treatment with antihypertensive medications does not necessarily translate into adequate BP control. A substantial proportion of adults receiving antihypertensive therapy remain above recommended BP targets [3]. This persistent treatment-control gap highlights the importance of identifying factors associated with uncontrolled BP among treated adults.

Nationally representative data demonstrate important changes in BP control in the United States over time. Analyses of the National Health and Nutrition Examination Survey (NHANES) showed that BP control improved substantially from 1999-2000 through 2007-2008, remained relatively stable through 2013-2014, and subsequently declined by 2017-2018 [5]. More recent evidence indicates that the earlier decline did not continue through 2021-2023; nevertheless, a substantial proportion of adults receiving antihypertensive therapy remained uncontrolled [6]. Changes in antihypertensive prescribing have also not eliminated this treatment-control gap. Although multiple antihypertensive classes and combination regimens are available, uncontrolled BP remains common among treated adults, including among those receiving monotherapy [7]. These patterns point to persistent challenges across the hypertension control cascade, including treatment initiation and intensification, continuity of care, and long-term maintenance of BP control [8,9].

The factors associated with uncontrolled BP among adults receiving antihypertensive therapy are multifactorial and extend beyond medication use alone [10]. Older age and obesity have been associated with poorer BP control, while resistant hypertension represents an important high-risk phenotype among treated adults [11,12]. Healthcare utilization may also influence hypertension management; adults with hypertension who do not have regular healthcare encounters are less likely to achieve effective treatment and BP control [10,13]. In addition, substantial racial and ethnic disparities in hypertension burden and BP control have been documented in the United States, with non-Hispanic Black adults experiencing a particularly high burden of hypertension [14,15]. Social determinants of health further intersect with these clinical and demographic factors. Lower educational attainment, socioeconomic disadvantage, lack of health insurance, and barriers to healthcare access have been associated with adverse hypertension outcomes and poorer BP control [16-18]. Collectively, these findings suggest that uncontrolled BP among treated adults reflects the interaction of demographic, socioeconomic, behavioral, clinical, treatment-related, and healthcare-access factors rather than pharmacological treatment alone.

Failure to achieve BP control despite antihypertensive treatment may also have important long-term prognostic implications. Previous NHANES-linked mortality research found that uncontrolled hypertension, including treated but uncontrolled hypertension, was associated with increased risks of all-cause and cardiovascular mortality [19]. Apparent treatment-resistant hypertension has similarly been associated with increased cardiovascular mortality among U.S. adults [12]. In addition, observational evidence suggests that maintaining BP control over time among adults receiving antihypertensive therapy may be associated with more favorable cardiovascular outcomes [20]. These findings are consistent with evidence from large meta-analyses demonstrating that pharmacological BP lowering reduces the risk of major cardiovascular events and mortality [1,4]. However, much of the nationally representative evidence linking treated but uncontrolled hypertension with mortality has been derived from earlier NHANES cohorts, particularly NHANES III, and may not fully reflect more contemporary patterns of antihypertensive treatment, cardiovascular risk, and BP control [19].

Previous studies have separately examined reasons for uncontrolled BP [10], antihypertensive treatment patterns [7], healthcare utilization [13], racial and ethnic disparities [14,15], social determinants of BP control [16-18], and the mortality implications of uncontrolled or resistant hypertension [12,19]. However, contemporary nationally representative evidence simultaneously characterizing factors associated with uncontrolled BP specifically among adults receiving antihypertensive therapy and evaluating its association with subsequent mortality remains limited. Addressing these questions within a contemporary treated population may help identify groups at increased risk of inadequate BP control while clarifying the prognostic significance of remaining uncontrolled despite treatment. Therefore, this study used NHANES 2013-2018 data linked to the National Center for Health Statistics Mortality File to identify demographic, socioeconomic, behavioral, clinical, treatment-related, and healthcare-access factors associated with uncontrolled BP among U.S. adults receiving antihypertensive therapy and to evaluate the association of uncontrolled BP with subsequent heart-disease and all-cause mortality.

## Materials and methods

Study design and data source

This retrospective cohort study used NHANES 2013 to 2018 data linked to the 2019 NCHS public use mortality file [21]. NHANES uses a complex, multistage probability sampling design to represent the civilian, noninstitutionalized United States population and includes interview, examination, and laboratory data. Relevant files were merged by the unique participant identifier, SEQN. Mortality status was determined using MORTSTAT, and follow-up through December 31, 2019, was calculated from PERMTH_EXM, the number of person-months from the examination to death or censoring, and converted to years [22]. Because the public use mortality file does not provide a consistent category for total cardiovascular mortality across the study cycles, heart disease mortality was used as the cause-specific mortality outcome.

Study population

The initial pooled NHANES 2013-2018 sample contained 29,400 participants. Participants younger than 18 years were excluded, leaving 17,961 adults. A further 78 participants who were not eligible for mortality linkage were excluded, leaving 17,883 mortality linkage-eligible adults. The study population was then restricted to adults who reported currently taking prescribed medication for hypertension, identified from the NHANES blood pressure questionnaire. This restriction resulted in 4,789 adults receiving antihypertensive therapy. Five pregnant participants were subsequently excluded, leaving 4,784 eligible treated adults. Complete case analysis was used for the prespecified variables required for the primary analyses. A total of 1,473 participants had incomplete data for one or more required variables and were excluded. The final analytic sample therefore consisted of 3,311 participants. After application of the combined NHANES examination weights, this sample represented an estimated 42,895,551 adults in the United States population, as shown in Figure 1 below.

**Figure 1:**
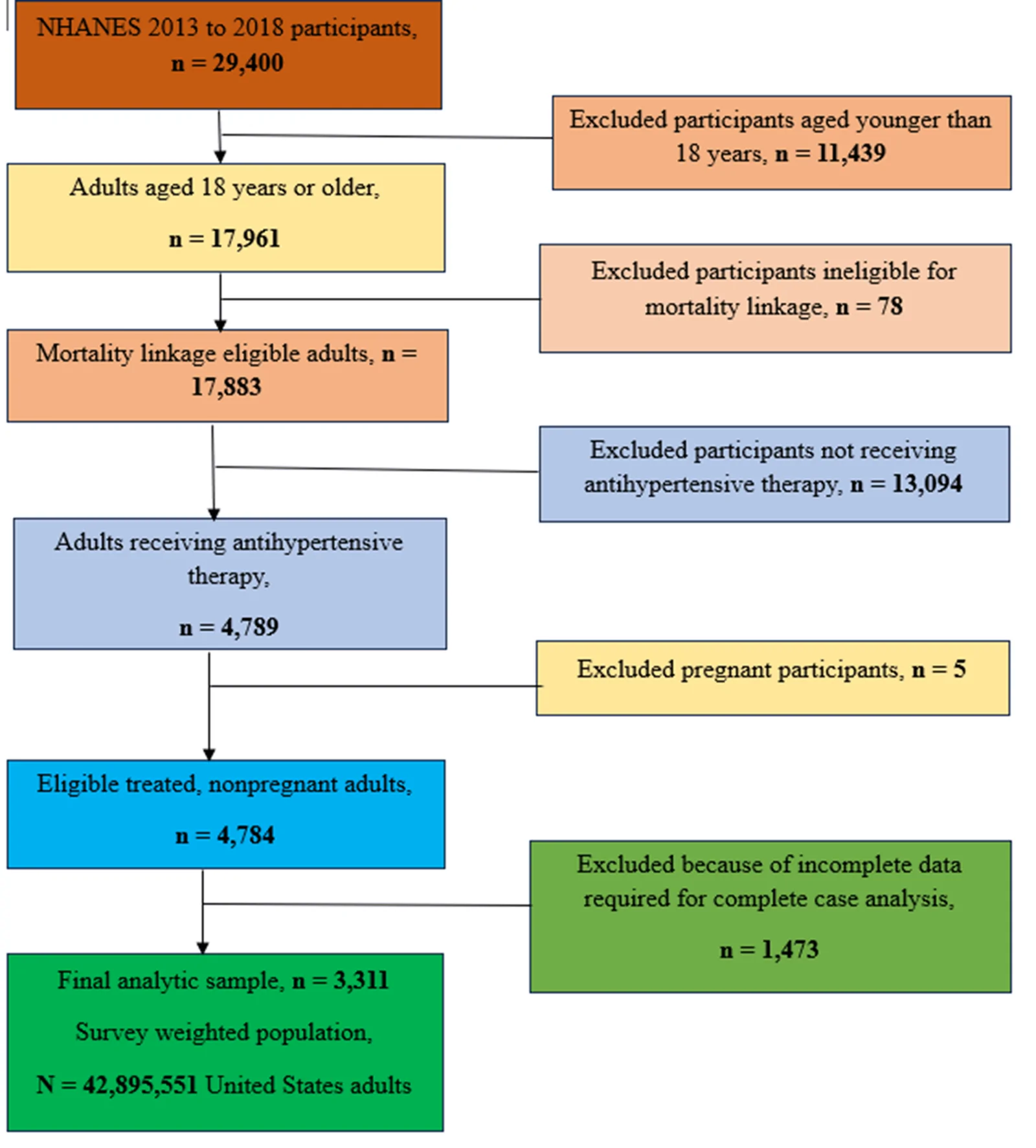
NHANES 2013-2018 Study Population Selection

Variables and measures

The primary outcome for the cross-sectional analysis was uncontrolled blood pressure. Up to four blood pressure readings were obtained during the NHANES mobile examination center examination. When at least two readings were available, the first reading was excluded and the available second to fourth readings were averaged. When only one reading was available, that reading was used. Uncontrolled blood pressure was defined as mean systolic blood pressure of at least 130 mmHg or mean diastolic blood pressure of at least 80 mmHg. We applied this threshold to all 2013-2018 NHANES cycles to assess blood pressure control consistently across the study period using the 2017 ACC/AHA definition [2]. Current antihypertensive therapy was defined by self-reported current use of prescribed medication for hypertension.

The mortality outcomes were all-cause and heart disease mortality, determined from the NHANES Linked Mortality File. Follow-up was calculated from the NHANES examination to death or the end of mortality follow-up. Heart disease deaths were identified using the public-use leading cause of death recode. For the cause-specific Cox analysis, deaths from other causes were censored at their observed follow-up time.

Covariates were selected based on clinical relevance and availability across the 2013-2014, 2015-2016, and 2017-2018 NHANES cycles. Demographic and socioeconomic variables included age, sex, race and ethnicity, education, family income-to-poverty ratio, and marital status. Race and ethnicity were categorized as Mexican American, other Hispanic, non-Hispanic White, non-Hispanic Black, non-Hispanic Asian, or other race. Education was grouped as less than high school, high school or GED, and college or higher. Marital status was categorized as married or living with a partner, widowed, divorced or separated, or never married.

Clinical and behavioral covariates included body mass index, smoking status, alcohol use, diabetes, chronic kidney disease, non-HDL cholesterol, prevalent cardiovascular disease, and depressive symptoms. Smoking was categorized as never, former, or current. Alcohol use during the previous 12 months was harmonized across survey cycles to account for questionnaire changes. Diabetes was defined by self-reported diagnosis, insulin or diabetes medication use, or glycated hemoglobin of at least 6.5%. Chronic kidney disease was defined as estimated glomerular filtration rate below 60 mL/min/1.73 m^2^ or urine albumin to creatinine ratio of at least 30 mg/g, with estimated glomerular filtration rate calculated using the 2021 CKD EPI creatinine equation [23]. Non-HDL cholesterol was calculated as total cholesterol minus HDL cholesterol.

Prevalent cardiovascular disease was defined by self-reported congestive heart failure, coronary heart disease, angina, myocardial infarction, or stroke. Depressive symptoms were defined as a Patient Health Questionnaire 9 score of at least 10 [24]. Health insurance coverage and having a routine place for healthcare were included as healthcare-related covariates. Treatment intensity was measured as the number of antihypertensive medication categories identified in the NHANES prescription medication files. We searched reported drug names for specified ingredients and classified them as ACE inhibitors, angiotensin receptor blockers, beta blockers, calcium channel blockers, thiazide or thiazide-like diuretics, or other antihypertensives. Each category was counted once per participant. Fixed-dose combination products contributed to more than one category when their recorded names identified ingredients from different categories.

Missing data

Missing data were handled using complete case analysis because missingness for individual covariates was generally modest and some questionnaire variables were subject to NHANES skip patterns. This approach also maintained a consistent analytic sample across the prespecified models without introducing assumptions required for imputation. Among the 4,784 eligible participants, missingness was highest for depression, 576 (12.04%), family income to poverty ratio, 552 (11.54%), alcohol use, 506 (10.58%), non-HDL cholesterol, 413 (8.63%), chronic kidney disease, 410 (8.57%), blood pressure measurements, 180 (3.76%) for each case, diabetes, 362 (7.57%), and body mass index, 275 (5.75%). Missingness for the remaining model covariates was below 1%. Overall, 1,473 (30.79%) participants had incomplete data for at least one required variable and were excluded, leaving 3,311 (69.21%) participants for analysis. Potential selection bias from complete case exclusion was considered when interpreting the findings.

Statistical analysis

Analyses were performed in Stata version 18 (StataCorp LLC, College Station, TX) using procedures that accounted for the NHANES complex survey design, including strata, clusters, and examination weights. Because three two-year cycles were combined, the six-year examination weight was calculated as WTMEC2YR divided by three. Continuous variables were summarized using survey-weighted means and standard deviations, while categorical variables were presented as unweighted counts and survey-weighted percentages. Comparisons by blood pressure control status used survey-adjusted t tests for continuous variables and survey-adjusted chi-square tests for categorical variables. Survey-weighted prevalence of uncontrolled blood pressure was also estimated by NHANES cycle with 95% confidence intervals.

Factors associated with uncontrolled blood pressure were examined using survey-weighted multivariable logistic regression, adjusting for the prespecified demographic, socioeconomic, behavioral, clinical, healthcare, and treatment covariates. Results were reported as adjusted odds ratios with 95% confidence intervals. Multicollinearity was assessed using variance inflation factors, which ranged from 1.03 to 3.17, with a mean of 1.45.

Associations of uncontrolled blood pressure with all-cause and heart disease mortality were examined using survey-weighted Cox proportional hazards regression and reported as adjusted hazard ratios with 95% confidence intervals. The all-cause mortality model included the full prespecified covariate set. Because only 86 heart disease deaths occurred, a parsimonious model was used to reduce model instability and included age, sex, race and ethnicity, body mass index, smoking, diabetes, chronic kidney disease, prevalent cardiovascular disease, and number of antihypertensive medication classes. Deaths from other causes were censored at their observed follow-up time in the heart disease model. The proportional hazards assumption was assessed using the global scaled Schoenfeld residual test in an unweighted refit of the all-cause mortality Cox model. The test did not indicate an overall violation (χ²(25) = 26.59, p = 0.377). All tests were two-sided, with P < 0.05 considered statistically significant.

Ethical considerations

This study was a secondary analysis of publicly available, de-identified NHANES data and the public-use NCHS Linked Mortality File that involved no direct participant contact. NHANES data collection was approved by the NCHS Research Ethics Review Board under Protocol #2011-17, with informed consent obtained from participants.

## Results

Table 1 below presents the demographic, socioeconomic, clinical, behavioral, and healthcare characteristics of adults receiving antihypertensive therapy according to blood pressure control status.

**Table 1:** Baseline characteristics of adults receiving antihypertensive therapy by blood pressure control status, NHANES 2013-2018 Continuous variables are presented as survey-weighted means (SD). Categorical variables are presented as estimated US population counts (column percentages). Group comparisons for continuous variables used survey-adjusted t tests, reported as equivalent F statistics. Group comparisons for categorical variables used survey-adjusted chi-square tests, reported as design-based F statistics. Abbreviations: BP, blood pressure; SD, standard deviation; GED, General Educational Development; BMI, body mass index; CKD, chronic kidney disease; CVD, cardiovascular disease; PHQ-9, Patient Health Questionnaire 9; HDL, high-density lipoprotein; MEC, mobile examination center. (-) intentionally left blank. The asterisk(*) denotes significance at 0.05.

Characteristic	Controlled BP, weighted N = 18,474,916	Uncontrolled BP, weighted N = 24,420,635	Test statistic	p-value
Age, years, mean ± SD	61.01 ± 11.59	62.15 ± 13.04	F = 1.70	0.199
Income-to-poverty ratio, mean ± SD	3.18 ± 1.54	2.93 ± 1.65	F = 9.36	0.004*
Body mass index, kg/m², mean ± SD	31.88 ± 6.71	31.87 ± 7.63	F = 0.00	0.992
Mean systolic BP, mmHg, mean ± SD	116.22 ± 8.95	143.28 ± 15.86	F = 2877.52	<0.001*
Mean diastolic BP, mmHg, mean ± SD	65.52 ± 9.63	74.77 ± 15.89	F = 186.69	<0.001*
Non-HDL cholesterol, mg/dL, mean ± SD	132.24 ± 43.75	138.32 ± 44.22	F = 6.71	0.013*
Number of antihypertensive medication classes, mean ± SD	1.83 ± 0.88	1.82 ± 0.97	F = 0.04	0.847
Gender, n (%)	-	-	F = 0.76	0.389
Male	8,360,330 (45%)	11,462,519 (47%)	-	-
Female	10,114,586 (55%)	12,958,116 (53%)	-	
Race and ethnicity, n (%)	-	-	F = 6.72	<0.001*
Mexican American	940,968 (5%)	1,154,165 (5%)	-	-
Other Hispanic	618,344 (3%)	1,171,697 (5%)	-	-
Non-Hispanic White	13,842,025 (75%)	16,552,490 (68%)	-	-
Non-Hispanic Black	1,947,728 (11%)	3,697,442 (15%)	-	-
Non-Hispanic Asian	536,267 (3%)	1,089,667 (4%)	-	-
Other race	589,584 (3%)	755,174 (3%)	-	-
Education, n (%)	-	-	F = 6.34	0.003*
Less than high school	2,232,538 (12%)	3,738,141 (15%)	-	-
High school graduate/GED	4,567,444 (25%)	6,971,474 (29%)	-	-
College or higher	11,674,934 (63%)	13,711,020 (56%)	-	-
Marital status, n (%)	-	-	F = 0.67	0.509
Married/living with partner	11,649,297 (63%)	16,004,505 (65%)	-	-
Widowed/divorced/separated	5,430,483 (29%)	6,800,869 (28%)	-	-
Never married	1,395,136 (8%)	1,615,261 (7%)	-	-
Smoking status, n (%)	-	-	F = 0.65	0.522
Never	9,058,305 (49%)	12,556,349 (51%)	-	-
Former	6,343,263 (34%)	8,293,967 (34%)	-	-
Current	3,073,348 (17%)	3,570,319 (15%)	-	-
Alcohol use in past 12 months, n (%)	-	-	F = 2.71	0.107
Yes	12,946,140 (70%)	16,046,352 (66%)	-	-
No	5,528,776 (30%)	8,374,283 (34%)	-	-
Diabetes, n (%)	-	-	F = 0.01	0.935
Yes	6,006,959 (33%)	7,998,930 (33%)	-	-
No	12,467,957 (67%)	16,421,705 (67%)	-	-
Chronic kidney disease, n (%)	-	-	F = 3.91	0.054
Yes	4,853,689 (26%)	7,771,135 (32%)	-	-
No	13,621,227 (74%)	16,649,500 (68%)	-	-
Prevalent cardiovascular disease, n (%)	-	-	F = 1.62	0.210
Yes	4,335,019 (23%)	5,116,682 (21%)		
No	14,139,897 (77%)	19,303,953 (79%)	-	-
Health insurance, n (%)	-	-	F = 3.38	0.073
Yes	17,542,722 (95%)	22,667,652 (93%)	-	-
No	932,194 (5%)	1,752,983 (7%)	-	-
Routine place for healthcare, n (%)	-	-	F = 1.68	0.202
Yes	17,983,800 (97%)	23,508,740 (96%)	-	-
No	491,116 (3%)	911,895 (4%)	-	-
Depressive symptoms, PHQ-9 ≥10, n (%)	-	-	F = 0.034	0.854
Yes	2,054,950 (11%)	2,640,812 (11%)	-	-
No	16,419,966 (89%)	21,779,823 (89%)	-	-

Adults with uncontrolled BP had a lower income-to-poverty ratio, lower educational attainment, and higher non-HDL cholesterol. Blood pressure control status also differed by race and ethnicity. Age, sex, BMI, marital status, smoking, alcohol use, diabetes, prevalent CVD, healthcare access, and treatment intensity were similar between the groups. CKD was more common among adults with uncontrolled BP, but the difference was not statistically significant. Depressive symptoms appeared similar and were not statistically significant.

Table 2 below shows the factors associated with uncontrolled blood pressure among adults receiving antihypertensive therapy.

**Table 2:** Factors associated with uncontrolled blood pressure among adults receiving antihypertensive therapy, NHANES 2013-2018 Adjusted odds ratios and 95% confidence intervals were estimated using survey-weighted multivariable logistic regression. The model accounted for NHANES examination weights, strata, and clusters. Continuous variable estimates represent the change in odds for each one-unit increase. Non-Hispanic White, male, college education or higher, married or living with a partner, never smoker, no alcohol use, no diabetes, no CKD, no prevalent CVD, no health insurance, no routine place for healthcare, and no depressive symptoms were the reference categories where applicable. Abbreviations: OR, odds ratio; CI, confidence interval; BMI, body mass index; CKD, chronic kidney disease; CVD, cardiovascular disease; HDL, high-density lipoprotein. (-) intentionally left blank. The asterisk(*) denotes significance at 0.05.

Characteristic	Adjusted OR	95% CI	p-value
Age, per 1-year increase	1.01	1.00 to 1.03	0.051
Female vs male	0.84	0.71 to 1.00	0.044*
Race and ethnicity	-	-	-
Mexican American vs Non-Hispanic White	0.89	0.65 to 1.22	0.473
Other Hispanic vs Non-Hispanic White	1.50	1.08 to 2.07	0.016*
Non-Hispanic Black vs Non-Hispanic White	1.63	1.29 to 2.05	<0.001*
Non-Hispanic Asian vs Non-Hispanic White	1.68	1.15 to 2.45	0.008*
Other race vs Non-Hispanic White	1.10	0.66 to 1.83	0.714
Education	-	-	-
Less than high school vs college or higher	1.24	0.99 to 1.56	0.064
High school/GED vs college or higher	1.25	1.01 to 1.55	0.042*
Income-to-poverty ratio, per 1-unit increase	0.92	0.84 to 1.00	0.042*
Marital status	-	-	-
Widowed/divorced/separated vs married/living with partner	0.80	0.63 to 1.01	0.058
Never married vs married/living with partner	0.79	0.54 to 1.13	0.192
BMI, per 1-kg/m² increase	1.01	0.99 to 1.02	0.518
Smoking status	-	-	-
Former vs never	0.93	0.73 to 1.17	0.519
Current vs never	0.83	0.60 to 1.14	0.241
Alcohol use, yes vs no	0.96	0.75 to 1.24	0.773
Diabetes, yes vs no	0.92	0.72 to 1.17	0.489
CKD, yes vs no	1.23	0.95 to 1.60	0.114
Non-HDL cholesterol, per 1-mg/dL increase	1.004	1.001 to 1.006	0.009*
Prevalent CVD, yes vs no	0.79	0.64 to 0.98	0.031*
Health insurance, yes vs no	0.74	0.48 to 1.14	0.165
Routine place for healthcare, yes vs no	0.83	0.49 to 1.41	0.479
Depressive symptoms, yes vs no	0.98	0.65 to 1.47	0.919
Number of antihypertensive medication classes, per 1-class increase	0.98	0.90 to 1.08	0.736

Female participants had lower adjusted odds of uncontrolled BP than male participants (adjusted OR 0.84, 95% CI, 0.71-1.00). Compared with non-Hispanic White adults, higher odds were observed among other Hispanic adults (adjusted OR 1.50, 95% CI, 1.08-2.07, non-Hispanic Black adults adjusted OR 1.63, 95% CI, 1.29-2.05, and non-Hispanic Asian adults adjusted OR 1.68, 95% CI, 1.15-2.45). High school or GED education was associated with higher odds than college education or higher. Each one-unit increase in the income-to-poverty ratio was associated with lower odds (adjusted OR 0.92, 95% CI, 0.84-1.00). Higher non-HDL cholesterol was associated with a small increase in the odds of uncontrolled BP, while prevalent CVD was associated with lower odds. BMI, smoking, alcohol use, diabetes, CKD, healthcare access, depressive symptoms, and number of antihypertensive medication classes were not statistically associated with uncontrolled BP.

Table 3 below presents the adjusted association between uncontrolled blood pressure and all-cause mortality.

**Table 3:** Association of uncontrolled blood pressure with all-cause mortality among adults receiving antihypertensive therapy, NHANES 2013-2018 Adjusted hazard ratios were estimated using survey-weighted Cox proportional hazards regression accounting for the NHANES complex survey design. Abbreviations: HR, hazard ratio; CI, confidence interval; BP, blood pressure; BMI, body mass index; CKD, chronic kidney disease; CVD, cardiovascular disease; HDL, high-density lipoprotein. (-) intentionally left blank. The asterisk(*) denotes significance at 0.05.

Characteristic	Adjusted HR	95% CI	p-value
Uncontrolled vs controlled BP	1.24	0.87 to 1.78	0.233
Age, per 1-year increase	1.08	1.06 to 1.11	<0.001*
Female vs male	0.73	0.54 to 0.98	0.038*
Race and ethnicity	-	-	-
Mexican American vs Non-Hispanic White	0.62	0.38 to 1.00	0.049*
Other Hispanic vs Non-Hispanic White	0.47	0.29 to 0.75	0.002*
Non-Hispanic Black vs Non-Hispanic White	0.62	0.46 to 0.86	0.004*
Non-Hispanic Asian vs Non-Hispanic White	0.34	0.14 to 0.82	0.017*
Other race vs Non-Hispanic White	0.76	0.34 to 1.69	0.490
Education	-	-	-
Less than high school vs college or higher	0.60	0.40 to 0.89	0.013*
High school/GED vs college or higher	0.72	0.52 to 1.00	0.050*
Income-to-poverty ratio, per 1-unit increase	0.76	0.63 to 0.91	0.004*
Marital status	-	-	-
Widowed/divorced/separated vs married/living with partner	1.56	1.10 to 2.21	0.014*
Never married vs married/living with partner	0.98	0.44 to 2.21	0.961
BMI, per 1-kg/m² increase	1.01	0.99 to 1.04	0.344
Smoking status	-	-	-
Former vs never	1.11	0.73 to 1.70	0.618
Current vs never	1.85	1.22 to 2.80	0.005*
Alcohol use, yes vs no	0.94	0.71 to 1.25	0.674
Diabetes, yes vs no	1.38	0.89 to 2.13	0.142
CKD, yes vs no	1.65	1.26 to 2.16	0.001*
Non-HDL cholesterol, per 1-mg/dL increase	0.999	0.995 to 1.003	0.618
Prevalent CVD, yes vs no	1.58	1.07 to 2.33	0.023*
Health insurance, yes vs no	0.43	0.21 to 0.89	0.023*
Routine place for healthcare, yes vs no	4.07	1.31 to 12.57	0.016*
Depressive symptoms, yes vs no	1.00	0.45 to 2.21	0.991
Number of antihypertensive medication classes, per 1-class increase	0.98	0.82 to 1.18	0.843

Among 3,311 participants, median recorded follow-up was 3.83 years (IQR 2.33-5.25). The all-cause mortality Cox model included 3,309 participants with positive follow-up time, 291 deaths, and 12,712.5 unweighted person-years. Uncontrolled BP was not statistically associated with all-cause mortality after adjustment (HR 1.24, 95% CI, 0.87-1.78; p=0.233). Mortality hazard increased with age and was higher among current smokers, adults with CKD, adults with prevalent CVD, and adults who were widowed, divorced, or separated. Female participants had a lower mortality hazard than male participants. Lower hazards were also observed among Mexican American, other Hispanic, non-Hispanic Black, and non-Hispanic Asian adults compared with non-Hispanic White adults. A higher income-to-poverty ratio and health insurance coverage were associated with lower mortality hazards. Less than high school education was associated with a lower hazard than college education or higher. Participants reporting a routine place for healthcare had a higher adjusted hazard of all-cause mortality (HR 4.07, 95% CI, 1.31-12.57; p=0.016). The wide confidence interval indicates substantial uncertainty, and this association may reflect differences in underlying health status, residual confounding, or reverse causation rather than an effect of routine healthcare access. High school or GED education had a p-value of 0.050 and did not fall below the prespecified significance threshold.

Table 4 below presents the adjusted association between uncontrolled blood pressure and heart disease mortality.

**Table 4:** Association of uncontrolled blood pressure with heart disease mortality among adults receiving antihypertensive therapy, NHANES 2013-2018 Adjusted hazard ratios and 95% confidence intervals were estimated using a survey-weighted cause-specific Cox proportional hazards model that accounted for NHANES examination weights, strata, and clusters. Heart disease mortality was identified by leading cause of death code 001 in the public-use Linked Mortality File. Deaths from other causes were censored at their observed follow-up time. Because 86 heart disease deaths occurred, the model was restricted to age, sex, race and ethnicity, BMI, smoking, diabetes, CKD, prevalent CVD, and number of antihypertensive medication classes. Non-Hispanic White, male, and never smoker were the reference categories for race and ethnicity, sex, and smoking. HR, hazard ratio. CI, confidence interval. Abbreviations: BP, blood pressure; BMI, body mass index; CKD, chronic kidney disease; CVD, cardiovascular disease. (-) intentionally left blank. The asterisk(*) denotes significance at 0.05.

Characteristic	Adjusted HR	95% CI	p-value
Uncontrolled vs controlled BP	0.97	0.51 to 1.84	0.933
Age, per 1-year increase	1.12	1.07 to 1.16	<0.001*
Female vs male	0.39	0.18 to 0.85	0.019*
Race and ethnicity	-	-	-
Mexican American vs Non-Hispanic White	0.76	0.24 to 2.46	0.641
Other Hispanic vs Non-Hispanic White	1.32	0.51 to 3.41	0.554
Non-Hispanic Black vs Non-Hispanic White	0.83	0.35 to 1.98	0.672
Non-Hispanic Asian vs Non-Hispanic White	0.51	0.14 to 1.82	0.290
Other race vs Non-Hispanic White	1.45	0.32 to 6.60	0.622
BMI, per 1-kg/m² increase	1.05	0.99 to 1.12	0.118
Smoking status	-	-	-
Former vs never	0.65	0.39 to 1.09	0.098
Current vs never	1.79	0.70 to 4.59	0.216
Diabetes, yes vs no	0.85	0.47 to 1.56	0.602
CKD, yes vs no	2.81	1.42 to 5.57	0.004*
Prevalent CVD, yes vs no	2.15	1.18 to 3.90	0.013*
Number of antihypertensive medication classes, per 1-class increase	0.93	0.72 to 1.19	0.554

Uncontrolled BP was not statistically associated with heart disease mortality, adjusted HR 0.97, 95% CI, 0.51-1.84, p = 0.933. Older age was associated with a higher heart disease mortality hazard. Female participants had a lower hazard than male participants, adjusted HR 0.39, 95% CI, 0.18-0.85, p = 0.019. CKD was associated with a higher hazard, adjusted HR 2.81, 95% CI, 1.42-5.57, p = 0.004, as was prevalent CVD, adjusted HR 2.15, 95% CI, 1.18-3.90, p = 0.013. Race and ethnicity, BMI, smoking, diabetes, and number of antihypertensive medication classes were not statistically associated with heart disease mortality in the parsimonious model.

Figure 2 below presents the survey-weighted prevalence of uncontrolled blood pressure across the three NHANES cycles.

**Figure 2:**
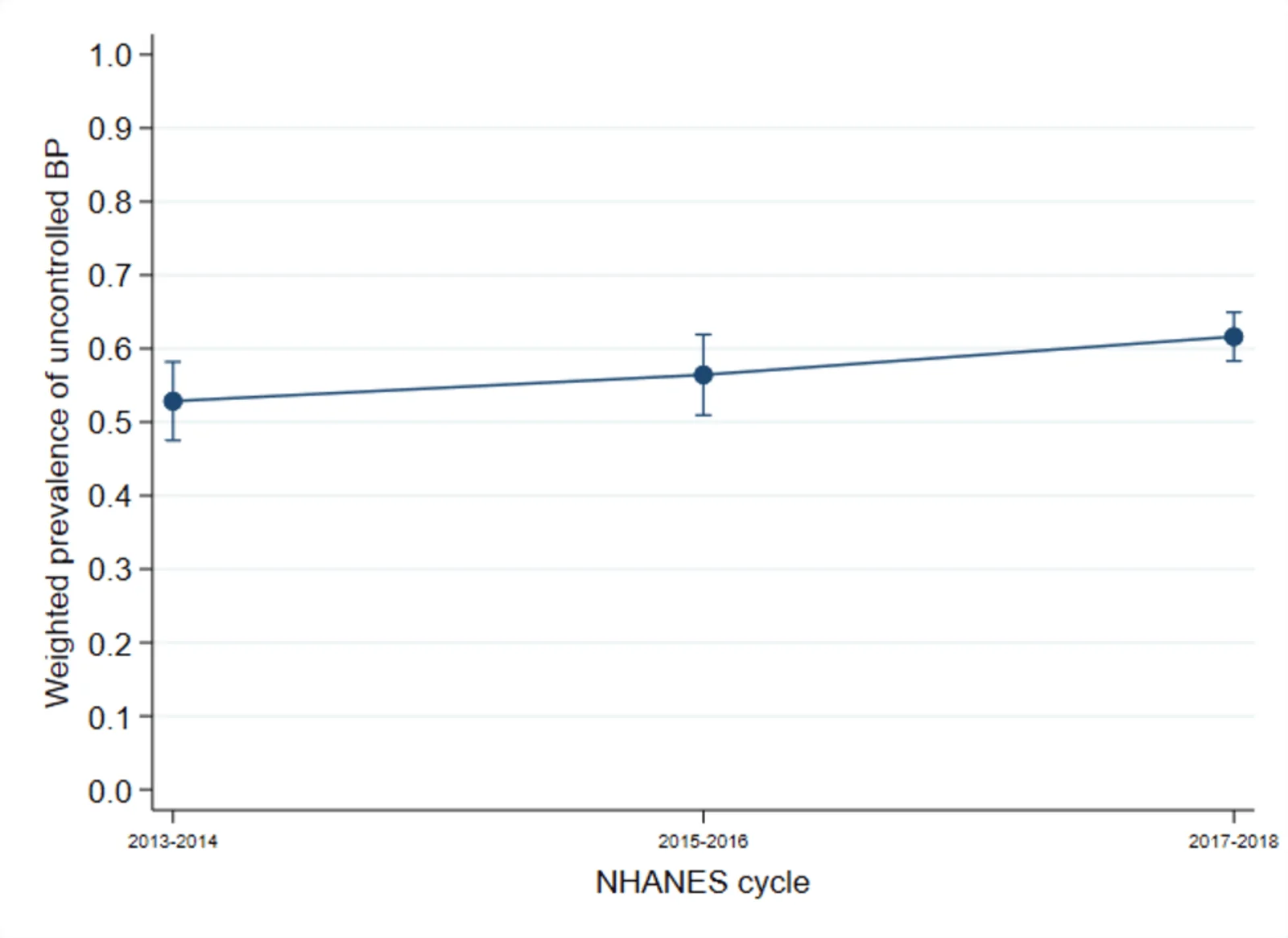
Survey-weighted prevalence of uncontrolled blood rressure by NHANES cycle, 2013-2018

The survey-weighted prevalence of uncontrolled BP was 52.8% (95% CI, 47.5-58.2%) in 2013-2014, 56.4% (95% CI, 50.9-61.9%) in 2015-2016, and 61.6% (95% CI, 58.3-64.9%) in 2017-2018. These percentages are survey-weighted estimates derived using NHANES examination weights and therefore do not correspond directly to unweighted participant counts. These are descriptive estimates; no formal test for trend was performed.

## Discussion

In this study, more than half of US adults receiving antihypertensive therapy had uncontrolled blood pressure. The weighted prevalence increased across the three NHANES cycles, although a formal trend test was not available. Higher odds of uncontrolled blood pressure were found among other Hispanic, non-Hispanic Black, and non-Hispanic Asian adults, while female participants had lower odds than male participants. High school/GED attainment, lower family income, and higher non-HDL cholesterol were associated with uncontrolled blood pressure. The education estimate in the separate all-cause mortality model was imprecise (adjusted HR 0.72, 95% CI, 0.52-1.00); because the models examined different outcomes, their estimates should not be compared directly. After adjustment, uncontrolled blood pressure was not statistically associated with all-cause or heart disease mortality. Age, current smoking, chronic kidney disease, and prevalent cardiovascular disease remained important markers of mortality risk.

The 2017 ACC/AHA guideline generally recommends a blood pressure target below 130/80 mmHg for treated adults, particularly those with cardiovascular disease or elevated cardiovascular risk [2]. It also emphasizes careful blood pressure measurement, lifestyle measures, appropriate drug combinations, and regular reassessment until control is achieved [2]. The high proportion above this target identifies an important gap between recommended care and measured blood pressure among treated adults. Blood pressure lowering trials indicate that effective treatment reduces cardiovascular events, although the size of benefit can vary with baseline pressure and clinical risk [1,4]. These findings support continued attention to accurate measurement and treatment review.

The racial and socioeconomic patterns agree with national studies showing persistent differences in hypertension treatment and control across population groups [8,14,15]. Social conditions can shape medication access, continuity of care, food choices, chronic stress, and opportunities for healthy activity [16-18]. These pathways may help explain why income and education remained related to control after adjustment. Higher odds among other Hispanic, non-Hispanic Black, and non-Hispanic Asian adults were consistent with reported disparities under the 2017 ACC/AHA threshold [14,15]. Insurance and a routine source of care were not significant, but most participants reported both forms of access. National cascade studies indicate that access does not always result in treatment intensification or control [8,9,13].

Higher non-HDL cholesterol had a small association with uncontrolled blood pressure and may reflect a broader cardiometabolic risk profile. BMI was not associated with control after adjustment, despite prior NHANES evidence relating excess body weight to blood pressure control [11]. The number of antihypertensive medication classes was also unrelated to control. Medication count does not capture dose, adherence, treatment duration, or the suitability of a drug combination. National prescribing data show considerable variation in monotherapy and combination patterns [7]. The lower odds among adults with prevalent cardiovascular disease should not be viewed as a protective effect. Closer monitoring after a cardiovascular diagnosis may contribute to this finding [8,9,13].

The lack of a significant mortality association differs from earlier NHANES III findings linking uncontrolled hypertension with all-cause and cardiovascular mortality [19]. Resistant hypertension and persistent poor control have also been associated with adverse cardiovascular outcomes in US cohorts [12,20]. Differences in population selection, blood pressure definition, follow-up, and outcome availability may account for the different estimates. This study included only adults receiving antihypertensive treatment. Blood pressure was assessed at a single NHANES examination visit, using the available readings from that visit as described in the Methods. Multiple readings at one visit cannot capture day-to-day variation or establish whether blood pressure remained uncontrolled over time. Follow-up was shorter for later cycles, and only 86 heart disease deaths were available, limiting precision. Chronic kidney disease and prevalent cardiovascular disease were strongly associated with heart disease mortality, consistent with their clinical importance as markers of vascular and target organ injury [12,20].

Strengths and limitations of the study

This study used standardized NHANES examination measurements and linked participants to prospective mortality data. Several limitations affect interpretation. Diagnoses and medication use were reported by participants and may have been misclassified. Complete-case analysis excluded 1,473 of 4,784 eligible treated adults (30.8%). If those excluded differed in blood pressure control or mortality risk, the associations may be biased; the direction and magnitude of that bias are unknown. Blood pressure was measured at a single examination visit, sometimes using multiple readings, and therefore cannot establish whether control was sustained over time. The analysis did not capture medication adherence, dose, or treatment changes, and reliable data on sodium intake, home or ambulatory blood pressure, and physical activity were unavailable. Residual confounding remains possible, particularly for the association between a routine place for healthcare and mortality. The heart-disease mortality model had only 86 events, so its adjusted estimates may be unstable despite the reduced covariate set. Blood pressure control factors were assessed cross-sectionally at one examination. Cause-specific mortality data were limited, and later NHANES cycles had shorter follow-up. Future studies should incorporate repeated blood pressure measurements, pharmacy records, detailed lifestyle data, and longer mortality follow-up.

## Conclusions

This study found that uncontrolled blood pressure was common among US adults receiving antihypertensive therapy and varied across racial, ethnic, educational, and income groups. Higher non-HDL cholesterol was associated with uncontrolled blood pressure, while the number of antihypertensive medication categories was not. We did not detect a statistically significant association between uncontrolled blood pressure and all-cause or heart disease mortality after adjustment; the confidence intervals, particularly for heart disease mortality, do not rule out an association. These findings should be interpreted in light of the single-visit blood pressure assessment, complete-case exclusion, and limited number of heart-disease deaths. Future studies with repeated blood pressure measurements, detailed medication and adherence data, and longer follow-up may provide more precise estimates.
